# Gut-on-Chip Models for Host–Microbiome Studies: A Systematic Review

**DOI:** 10.3390/microorganisms14092059

**Published:** 2026-09-15

**Authors:** Jennifer Redondo, Guillermo Garcia-Lainez, Verónica Martínez-Ríos, Empar Chenoll, Patricia Martorell

**Affiliations:** Archer Daniels Midland, Microbiome Research Center, Biopolis S.L. Parc Cientific Universitat de València, C/Catedrático Agustín Escardino Benlloch, 9, 46980 Paterna, Spain

**Keywords:** gut-on-chip, *in vitro* model, host–microbiome interactions, probiotic, postbiotic, biotic, intestinal microenvironment

## Abstract

As a central regulator of nutrient absorption, immune homeostasis and overall health, the gastrointestinal tract has become a major focus of biomedical research. However, developing *in vitro* models that accurately reproduce human gastrointestinal architecture and physiological conditions remains a major challenge. Gut-on-chip (GoC) systems, which integrate microfluidics with cell cultures, have emerged as a promising solution in the past decade. By recreating dynamic microenvironments that incorporate fluid flow, peristalsis-like mechanical stimulation and co-culture with microorganisms, GoC systems enable more physiologically relevant investigation of host–microbe and host–microbiome interactions. This systematic review provides a comprehensive overview of available GoC technology used in host–microbe research, including their structural and cellular components. A systematic search of PubMed, Embase and Google Scholar databases up to May 2026 identified forty-eight studies evaluating interactions between the host and probiotic strains, postbiotics, commensal microorganisms, pathogenic bacteria, fungi, viruses, or faecal-derived microbiota using GoCs. Common features, distinctive characteristics and application for modelling host–microbiome interactions and pathogenic infections are summarized. The available literature is characterized by heterogeneous study designs, variable microbiome compositions and analytical approaches, and limited cross-platform standardization, which should be considered when interpreting findings. Lastly, current limitations and future perspectives are discussed, highlighting the potential of GoC models to support biotic characterization and preclinical evaluation while underscoring the need for further validation and standardization.

## 1. Introduction

Gastrointestinal disorders are associated with a combination of factors, including altered motility, visceral hypersensitivity, impaired mucosal and immune function, disrupted intestinal microbiome and/or dysfunction of the central nervous system [1]. These conditions (i) have significant economic effects on healthcare systems and (ii) negatively affect quality of life. For example, a large-scale multinational study observed that between 20–40% of people in the world have gastrointestinal disorders [2].

Therefore, the role of the intestine is crucial in maintaining overall well-being. Despite its primary function being the extraction and absorption of nutrients from the food we consume, it also critically contributes to compound transport, metabolism and release of vital molecules. Additionally, the intestinal wall harbours the microbiome, which plays a key role in homeostasis by maintaining a bidirectional relationship with immune cells, safeguarding against pathogens [3] and producing antimicrobial substances [4] such as essential vitamins and neurologically active molecules like gamma-aminobutyric acid (GABA) and short-chain fatty acids (SCFAs) [5]. Alterations in the gut barrier and/or microbiome have been related to various gastrointestinal conditions, such as irritable bowel syndrome (IBS), small intestinal bacterial overgrowth (SIBO), diarrhoea or constipation, among others [6,7]. Hence, in recent years, there has been an increase in research and studies focused on ways in which nutrition can support human health. Several findings indicate that biotics such as probiotics and postbiotics and other nutritional interventions can modulate different cellular and physiological processes, which positively impact both the gut barrier and the microbiome [8,9]. However, understanding the underlying mechanisms driving these effects requires experimental models that accurately reproduce human physiology. Simulation and modelling of human pathological conditions, both *in vitro* and *in vivo* is often challenging due to intrinsic complexity. In this sense, evidence about the biotic interactions with both the intestine and microbiome has relied mainly on genomic or metagenomic analysis of human intestinal biopsies collected *in vivo* [10,11]. And although animal studies have been extensively used to study host–microbiome interactions and their contributions to the pathophysiology, several limitations should be pointed out, including high economical cost, ethical reasons, or differences between the composition of the animal and human microbiome. Existing *in vitro* models based on conventional cell culture methods, such as monolayer inserts, have been successfully used to investigate host–microbiome interactions. These cell culture systems offer several advantages, including their simplicity, low cost, high reproducibility and ease of manipulation. Nonetheless, these studies only provide information in the short term, as microbiota growth and metabolic activity progressively impair cell viability, ultimately leading to cellular damage and eventual death [12]. And although a certain degree of cell differentiation is achieved, cell morphology differs significantly from the native intestine structure. Gut organoids, 3D structures that mimic *in vitro* the cellular organization, physiology and heterogenicity of the gut, have already been employed for exploring the impact of different dietary components, such as β-glucans or catechins, on intestinal homeostasis or inflammatory responses to promote gut health [13]. Additionally, organoids can be generated from different regions of the gastrointestinal tract, including the small intestine and colon, thereby enabling the study of region-specific functions and responses relevant to gut health under both physiological and pathological conditions. Moreover, substantial progress has been made in the development of gut organoid models incorporating features such as integrated vascular networks [14], peristaltic-like activity [15], and physiologically relevant oxygen gradients that support anaerobic microbiota [16]. However, in the context of host–microbiome research, they can still face certain challenges similar to those observed in monolayer assays, particularly regarding the duration of co-culture with live microorganisms, which is generally limited to approximately one day in their spherical configuration [17].

As a complementary methodology to conventional cell cultures and organoid-based models, GoC systems have emerged in recent years as advanced miniaturized *in vitro* platforms that integrate microfluidics, microfabrication technologies, and compatible materials with biomedical applications to recreate key aspects of the intestinal microenvironment. By incorporating dynamic fluid flow, mechanical stimuli, and separate but interconnected compartments, these systems enable the investigation of gut physiological processes difficult to replicate in conventional cell culture and organoid models. Importantly, the controlled microenvironment provided by GoC platforms can support longer-term experiments and extended host–microbe co-cultures, facilitating the study of biological responses over time.

The first GoC consisted of a polydimethylsiloxane (PDMS) microdevice containing two microfluidic channels separated by a porous flexible membrane coated with extracellular matrix and lined by human intestinal epithelial Caco-2 cells [12]. The gut microenvironment was emulated by flowing fluid producing low shear stress and by cyclic strain mimicking peristaltic motions. Under these conditions, epithelial cell differentiation was accelerated with the formation of 3D villi-like structures and increased intestinal barrier function in comparison to Caco-2 cultured in inserts. This system enabled, for the first time, the analysis of host–microbiome interactions over longer periods by maintaining stable co-cultures for up to one week through regulation of medium flow [12]. The development of GoC technology was initially supported by public funding from agencies such as the National Institutes of Health (NIH), the Wyss Institute, the Defense Advanced Research Projects Agency (DARPA) or the U.S. Food and Drug Administration (FDA). This foundational work enabled the creation of several companies that have accelerated innovation in the field. As a result, research in GoC systems has expanded exponentially in recent years.

Current models recapitulate key structural features of the human intestine, including villi, crypts, and mucus, as well as essential functions such as absorption, secretion, barrier integrity, and metabolism. GoC is typically composed of at least one lineage of intestinal epithelial cells (established cell line or primary culture), but other cell lineages, such as vascular endothelial cells or immune cells, can be incorporated [18,19]. The reconstructed microenvironment in the chip may include fluid flow, tissue–tissue interface and also intestinal peristalsis [20]. In addition, microorganisms, including pathogens, probiotic strains and whole microbiome have been successfully co-cultivated in the GoC. By contrast, compared with probiotics, postbiotics have been less extensively investigated in these GoC systems. In this context, the use of GoC composed with, e.g., patients’ organoids, immune cells and microbiota can be potentially used to both reproduce relevant gastrointestinal disorders and to later evaluate the complex interplay between biotics and microbiome [21].

However, the heterogeneity of experimental variables among different GoC models developed to conduct microbiome studies, such as chip material and architecture, cell lineage selection, co-culture conditions and the analytical readouts, poses a challenge to their standardization. Several reviews have highlighted the key technological features of GoC systems and their diverse applications in host–microbiome research [22,23,24]. However, these studies have generally provided narrative overviews rather than systematically evaluating the methodologies employed across the field. Consequently, a systematic assessment of current microbiome-oriented GoC models could help identify common practices, methodological gaps, and opportunities for harmonization. To our knowledge, no systematic review has yet compiled and compared the shared features and differences of GoC technologies developed to study host–microbiome interactions, nor summarized their applications in modelling gastrointestinal disorders and pathogenic infections. Consequently, the aim of this systematic review was to summarize the current knowledge on microbiome studies using GoC technology. For this purpose, a comprehensive analysis of the different devices has been performed. Lastly, this review discusses the existing limitations and future trajectories of this emerging technological paradigm, highlighting its potential applicability in biotic characterization prior to clinical trials.

## 2. Materials and Methods

### 2.1. Inclusion and Exclusion Criteria

This systematic review was conducted using the guidelines outlined in the Preferred Reporting Items for Systematic Reviews and Meta-Analyses (PRISMA) statement [25].

Eligible studies were required to utilize a microfluidic GoC model designed to recapitulate key physiological features of the intestinal microenvironment, such as compartmentalized cell culture, controlled fluid flow, mechanical stimulation, and/or epithelial barrier function.

To meet the inclusion criteria, studies were required to include both a host-derived biological component and a microbial component. Host-derived components included intestinal epithelial cell lines, intestinal organoids and biopsies integrated into chip platforms. Conventional organoid cultures lacking microfluidic components were excluded. Eligible microbial components included probiotic strains, postbiotic preparations, commensal microorganisms, pathogenic bacteria, fungi, viruses, mixed microbial consortia, and faecal-derived microbiota. Accordingly, studies investigating direct host–microbe interactions, microbial modulation of host physiology, microbial pathogenicity, or the effects of microbiota-based interventions were included. Studies focused solely on chip engineering, materials characterization, fluid dynamics, or device validation without a biological host–microorganism interaction endpoint were excluded.

Original experimental research articles reporting primary data were eligible for inclusion, irrespective of the study design, provided that they employed an *in vitro* GoC platform meeting the predefined criteria. Reviews, systematic reviews, meta-analyses, editorials, commentaries, perspectives, letters without original data, conference abstracts, book chapters, study protocols, patents, dissertations, and other non-primary research publications were excluded. Preprints and other non-peer-reviewed publications were also excluded to ensure inclusion of studies that had undergone peer review.

Only articles published in peer-reviewed journals and available as full-text manuscripts were considered eligible. Publications written in English were included. Non-English publications were excluded due to practical limitations associated with screening and data extraction and to ensure consistent assessment of methodological quality across studies.

### 2.2. Search Strategy

A systematic review was conducted in PubMed, Embase, and Google Scholar databases between 22 April and 8 May 2026. The search and selection criteria were designed to obtain references encompassing all studies published in English from 2010 to 2026 that investigated host–microbiome interactions and pathogen infection dynamics using GoC technology.

The search strategy used for PubMed database included the following word combination: (“Gut-on-chip”[Text Word] OR “gut-on-a-chip”[Text Word] OR “Intestine on chip”[Text Word] OR “intestine-on-a-chip”[Text Word]) AND (“Microbiota”[Text Word] OR “probiotic”[Text Word] OR “Lactobacillus”[Text Word] OR “bifidobacter*”[Text Word] OR “anaerob*”[Text Word] OR “pathogen*”[Text Word] OR “consorti*”[Text Word] OR “probiotic*”[MeSH Terms]).

The search strategy for the Embase database included the following combination of terms: (“Gut-on-chip” OR “gut-on-a-chip” OR “Intestine on chip” OR “intestine-on-a-chip”) AND (“microbiota” OR “microbiotas” OR “microbiotas” OR “microbiotae” OR “probiotic*” OR “lactobacillus” OR “bifidobacter*” OR “anaerob*” OR “pathogen*” OR “consorti*”).

For the Google Scholar database, the following combination of terms was used: (“Gut-on-chip” OR “gut-on-a-chip” OR “Intestine on chip” OR “intestine-on-a-chip”) AND (“microbiota” OR “microbiotas” OR “microbiotae” OR “microbiota” OR “probiotic*” OR “lactobacillus” OR “bifidobacterium*” OR “anaerob*” OR “pathogen*” OR “consorti*”). The first 18 pages of Google Scholar results (180 records) were included in the systematic review, as the subsequently retrieved records were no longer relevant to the review topic and no additional potentially eligible studies were identified.

### 2.3. Selection Process

Studies identified through the search strategy were screened in two stages: (i) title and abstract screening and (ii) full-text assessment. Three reviewers (VMR, GGL, and JR) independently evaluated all records at both stages. Study inclusion decisions were determined by majority agreement. Whenever discrepancies occurred, the reviewers discussed the eligibility criteria and the study in question until a decision was reached. Following duplicate removal, 122 original peer-reviewed publications were assessed for eligibility.

### 2.4. Data Extraction and Synthesis

The characteristics summarized in this systematic review, including device material, fabrication method, number of channels, perfusion, stretch capability, oxygen control, cell sources, microbial composition, and application, were selected based on their relevance to the objectives of the review. The final set of variables and classification categories was established through discussion and agreement among the five authors. Extracted data were synthesized descriptively and used to generate the quantitative summaries presented in the Results section.

Data relevant to device design, biological components, and study applications were extracted from the included publications by JR. The extracted information was subsequently reviewed by GGL and VMR to verify accuracy and consistency with the original articles. Any uncertainties or discrepancies were discussed among the authors and resolved through consensus.

### 2.5. Risk of Bias (RoB) Analysis for Individual Studies

The three reviewers (VMR, GGL, JR) independently evaluated the risk of bias (RoB) of the included GoC studies. Because the studies included in this review were *in vitro* GoC experiments, an adapted version of SYRCLE’s RoB tool developed by De la Rosa González et al. [26] was used. While the original SYRCLE tool was developed for animal intervention studies, the version for *in vitro* assays assessment proposed by De la Rosa González et al. served as the foundation for our assessment and was further tailored to address methodological aspects relevant to GoC and microbiome research (see Table S1 in the Supplementary Materials). For each study, bias-related information was systematically assessed and summarized. Judgments were assigned as follows: “Yes” indicated a low risk of bias, “No” indicated a high risk of bias, and “Unclear” indicated insufficient reporting to allow a definitive assessment. Discrepancies between reviewers were resolved through discussion until consensus was reached.

## 3. Results

**Figure 1:**
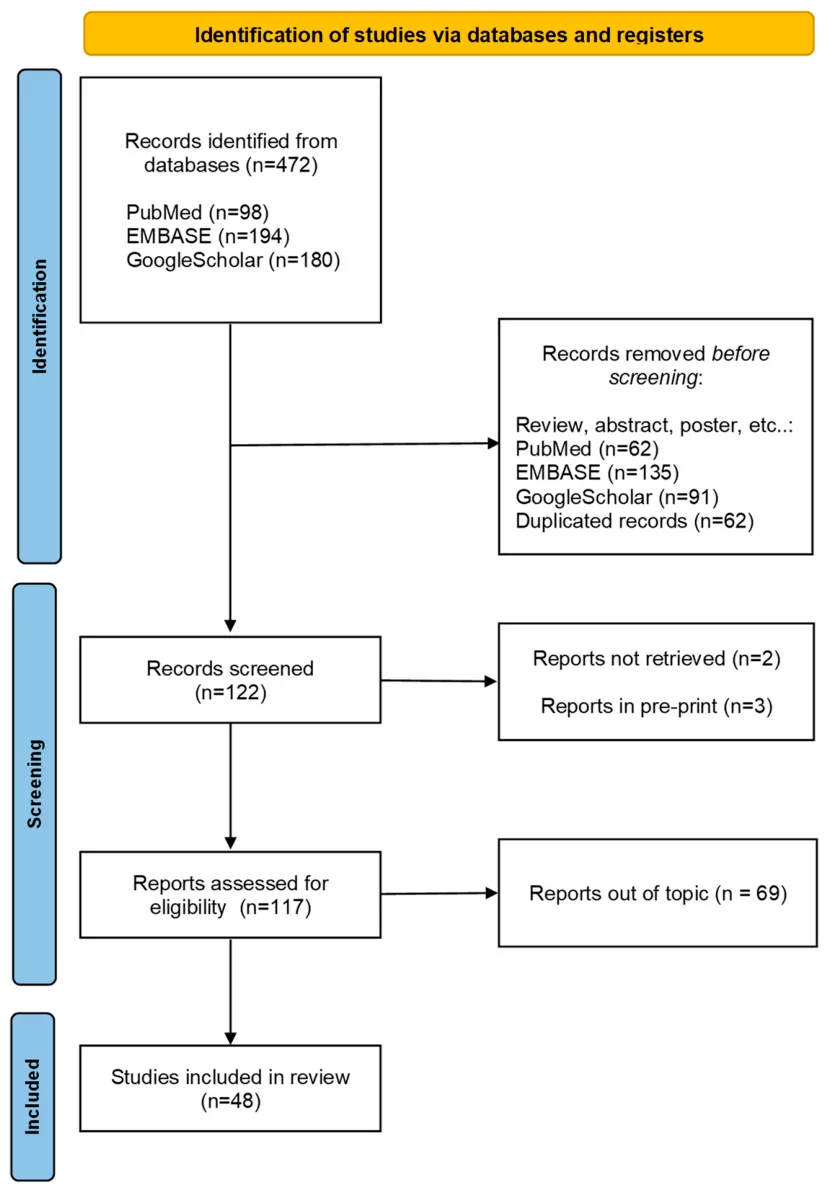
PRISMA flow chart of articles of the literature search, study selection process in the systematic review.

As shown in the PRISMA diagram (Figure 1), a total of 472 studies were identified from the initial database search across PubMed (*n* = 98), Embase (*n* = 194), and Google Scholar (*n* = 180). Prior to screening, 350 records were excluded, which comprised 62 duplicate records and 288 reports removed due to document type (reviews, abstracts, posters, etc.: 62 from PubMed, 135 from Embase, and 91 from Google Scholar). The remaining 122 records underwent title and abstract screening, resulting in the exclusion of 2 reports that could not be retrieved and 3 reports that were pre-prints. Out of the 117 reports assessed for full-text eligibility, 69 were excluded for being off-topic. Ultimately, 48 studies met all eligibility criteria and were included in the final review.

### 3.1. Risk of Bias (RoB) Assessment

Risk of bias was assessed for all 48 included studies using an adapted version of the SYRCLE Risk of Bias tool [26]. Detailed study-level assessments are provided in Table S2 of the Supplementary Materials.

Overall, the methodological quality of the included studies was generally acceptable, although several reporting limitations were identified. Due to the *in vitro* nature of the experimental models, almost all studies were rated as “No” for Items 1 and 8, which assess domains that are primarily applicable to *in vivo* studies. In contrast, most studies adequately reported the remaining methodological domains.

A limited number of studies showed deficiencies in specific reporting categories. Two studies [27,28] did not provide sufficient information regarding the bacterial strains or microbial communities employed (Item 3). Inadequate reporting of the microbiome intervention (Item 4) was identified in three studies [28,29,30]. Insufficient description of chip characteristics or experimental conditions (Item 5) was observed in four studies [29,30,31,32]. Additionally, two studies [28,33] lacked sufficient detail regarding the statistical analyses performed (Item 7).

Collectively, these findings indicate that the principal methodological concerns were related to incomplete reporting of experimental procedures and analytical methods rather than fundamental flaws in study design. Consequently, all eligible studies were retained and considered in the evidence synthesis, with the identified methodological limitations taken into account during data interpretation.

### 3.2. Comparison of Gut-on-Chip (GoC) Models Identified for Host–Microbiome Studies

The main characteristics of each GoC model, including chip architecture and material, perfusion, presence of cyclic stretch or the generation of oxygen gradients, are summarized in Table 1.

#### 3.2.1. Chip Materials, Fabric Methodology and Architecture

The most common material found in the GoC studies that include microbiome co-cultures was polydimethylsiloxane (PDMS; *n* = 36) followed by polycarbonate (PC; *n* = 5) and polystyrene (PS; *n* = 3). Other materials included were methacrylate and polybutylene terephthalate. All these materials share common features such as optical transparency and good biocompatibility, which are appropriate for cell culture and imaging. Among the distinctive features of PDMS are its intrinsic elasticity and high gas permeability, particularly for O_2_ and CO_2_, compared with PC and PS [34,35,36]. For microbiome studies in GoC systems, the intrinsic elasticity of PDMS enables the application of cyclic strain, thereby recapitulating the physiological peristaltic motions of the intestine. However, its high oxygen permeability constitutes both an advantage for maintaining host cell viability and a challenge for GoC models involving oxygen-sensitive microbiota, which require strict physiological oxygen gradients (see Section 3.2.3). In addition, the hydrophobic nature of PDMS may favour the initial adhesion of microorganisms to the chip surface. Nevertheless, in experiments involving both microbiome and hydrophobic drugs or metabolites, substantial absorption by PDMS may also occur, reducing their bioavailability and potentially affecting experimental outcomes.

Regarding the fabrication methodologies, soft lithography is the most commonly employed approach, particularly when PDMS is used as the material of choice (*n* = 31). In conventional soft lithography, a master mold is first fabricated using ultraviolet light and a photosensitive material to generate the desired microstructures. PDMS is then poured onto the master mold, cured, and peeled off to obtain the final microfluidic device [37]. Variants of this approach include 3D-printing-assisted soft lithography (*n* = 2) and micromilling-assisted soft lithography (*n* = 1), which follow the same replica-molding principle but employ alternative mold fabrication methods. Other chip fabrication methods include injection molding (*n* = 2), in which molten thermoplastic materials are injected into a mold cavity containing the desired microfluidic design. Finally, direct 3D printing (*n* = 2) and controlled/manual milling and laser-cutting techniques (*n* = 5) represent alternative fabrication strategies that do not require intermediate molds.

**Table 1:** Detailed overview of the main characteristics of the chips used in GoC in host–microbiome studies.

Material	Fabrication Method	Architecture	Channel Dimensions	Flow Conditions	Mechanical Stimulation	Oxygen Environment	Reference
Polydimethylsiloxane (PDMS)	Soft lithography	Two microchannels separated by a PDMS membrane	150 μm H × 1000 μm W	Unidirectional (40 μL/h)	Cyclic strain (10%, 0.15 Hz)	No specific control	[12]
Not stated	Not stated	Two channels separated by a polyamide semipermeable membrane and a mucus layer	10 cm × 6 cm	Unidirectional (6.5 mL/min and 2 mL/min)	No mechanical stimulation	Luminal anoxic medium (anaerobic chamber)	[38]
Polycarbonate (PC)	Controlled milling and laser-cut	Three microchannels separated by two semi-permeable membranes	4 mm W × 200 mm L × 0.5 mm H	Unidirectional (25 μL/min)	No mechanical stimulation	Luminal anoxic medium (nitrogen perfusion)	[39]
PDMS	Soft lithography	Two microchannels separated by a PDMS membrane	1 mm W × 10 mm L × 0.15 mm H	Unidirectional (40 μL/h)	Cyclic strain (10%, 0.15 Hz)	No specific control	[40]
PDMS	Soft lithography	Two microchannels separated by a PDMS membrane	1 mm W × 14 mm L × 0.2 mm H	Unidirectional (30 μL/h)	Cyclic strain (10%, 0.15 Hz)	No specific control	[34]
PDMS	Soft-lithography	Two microchannels separated by a PDMS membrane	1 mm W × 10 mm L × 0.2 mm H	Unidirectional (50 µL/h)	Cyclic strain (10%, 0.15 Hz)	No specific control	[41]
PDMS	Soft-lithography	Two microchannels separated by a PDMS membrane	1 mm W × 10 mm L × 0.2 mm H	Unidirectional (30 µL/h)	Cyclic strain (10%, 0.15 Hz)	No specific control	[35]
PC	Controlled milling and laser-cut	Three microchannels separated by two semi-permeable membranes	4 mm W × 200 mm L × 0.5 mm H	Unidirectional (25 μL/min)	No mechanical stimulation	Luminal anoxic medium (nitrogen perfusion)	[42]
PDMS	Soft-lithography	Two microchannels separated by a PDMS membrane	1 mm W × 10 mm L × 0.2 mm H	Unidirectional (65 μL/h)	Cyclic strain (10%, 0.15 Hz)	Luminal anoxic medium (anaerobic chamber)	[43]
Polystyrene (PS)	Injection molding	Two chambers separated by a polyethylene terephthalate membrane	Upper: 220 μL Lower: 110 μL	Unidirectional (25 μL/min upper and 50 μL/min lower)	No mechanical stimulation	No specific control	[44]
PDMS	Soft lithography	Two microchannels separated by a PDMS membrane	Upper: 500 μm H Lower: 200 μm H	Unidirectional (50 µL/h)	Cyclic strain (10%, 0.15 Hz)	Luminal anoxic medium (nitrogen perfusion)	[45]
PDMS	Soft lithography	Two microchannels separated by a PDMS membrane	Upper: 1000 μm W × 1000 μm H Lower: 1000 μm W × 200 μm H	Unidirectional (60 µL/h)	Not stated	No specific control	[46]
PDMS	Soft lithography	Two microchannels separated by 2 PDMS membranes	1000 μm W × 100 μm H × 30 mm L	Unidirectional (60 µL/h)	Cyclic strain (15%, 0.15 Hz).	No specific control	[18]
PDMS	Soft lithography	Two microchannels separated by a PDMS membrane	Not stated	Unidirectional (100 µL/h)	Cyclic strain (5%, 0.15 Hz)	Luminal anoxic medium (nitrogen perfusion, L-cysteine)	[47]
PDMS	Soft lithography	Two microchannels separated by a polytetrafluoroethylene membrane	1 mm W × 150 µm H × 1 cm L	Unidirectional (30 µL/h)	No mechanical stimulation	No specific control	[48]
PDMS	Soft lithography	Two microchannels separated by a PDMS membrane	Not stated	Unidirectional (60 µL/h)	Not stated	Luminal anoxic medium (anaerobic chamber)	[49]
PDMS	Soft lithography	Two channels separated by a PDMS membrane	1.5 mm W × 0.25 mm H × 8 mm L	Unidirectional (200 μL/h upper and 50 μL/h lower)	No mechanical stimulation	No specific control	[19]
PDMS	Soft lithography	Two channels separated by a PDMS membrane	Upper: 1 mm W × 1 mm H, Lower: 1 mm W × 0.2 mM H;	Unidirectional (60 µL/h)	Cyclic strain (10%, 0.15 Hz)	No specific control	[50]
PDMS	Soft lithography	Single channel	0.5 mm W × 0.35 mm H × 13 mm L	Unidirectional (120 µL/h)	No mechanical stimulation	No specific control	[27]
PDMS	Micromilling- assisted soft lithography	A central microchannel with an opened central chamber in which the insert is placed	Channel: 1 mm W × 1 mm H × 5 mm L; Chamber: 13 mm d × 5 mm H	Unidirectional (30 µL/h)	No mechanical stimulation	Luminal anoxic medium (nitrogen perfusion)	[51]
PDMS	Soft lithography	Three parallel microchannels separated by a hexagonal-shaped micropillar	500 μm W × 150 μm H × 10 mm L	Unidirectional (Flow rate not stated)	No mechanical stimulation	No specific control	[52]
PDMS	Soft lithography	Two microchannels separated by 2 PDMS membranes	1000 μm W × 100 μm H × 30 mm L	Unidirectional (60 μL/h)	Cyclic strain (15%, 0.17 Hz)	No specific control	[53]
PDMS	Soft lithography	Two-layer structure separated by a membrane	Upper: 6 mm d × 3 mm H Lower: 8 mm d × 0.3 mm H	Unidirectional (40 μL/min)	No mechanical stimulation	Luminal anoxic medium (nitrogen perfusion)	[28]
PDMS	Soft lithography	Two channels separated by a PDMS membrane	Upper: 500 μm H Lower: 200 μm H	Unidirectional (50 µL/h)	Cyclic strain (10%, 0.15 Hz)	Luminal anoxic medium (L-cysteine)	[54]
PDMS	Soft lithography	Two chambers separated by a PC membrane	2.75 mm R, 0.3 mm H	Unidirectional (60 µL/h)	No mechanical stimulation	No specific control	[55]
PDMS	Soft lithography	Two microchannels separated by a PDMS membrane	Not stated	Unidirectional (30 µL/h)	Cyclic strain (10%, 0.2 Hz)	No specific control	[21]
PDMS	Soft lithography	Two microchannels separated by a PDMS membrane	Upper: 1 W × 10 L × 0.5 H mm Lower: 1 W × 10 L × 0.2 H mm	Unidirectional (35 µL/h)	Cyclic strain (10%, 0.15 Hz)	Luminal anoxic medium (anaerobic jars)	[56]
PDMS	Soft lithography	Two microchannels separated by a PDMS membrane	Upper: 1 mm W × 1 mm H, Lower: 1 mm W × 0.2 mM H	Unidirectional (60 µL/h)	Cyclic strain (10%, 0.15 Hz)	No specific control	[57]
Methacrylate	3D printing-based fabrication protocol	Not stated	Not stated	Not stated	Not stated	No specific control	[29]
Polybutylene terephthalate	Injection molding	Two chambers separated by a PET membrane	Upper 290 μL Lower: 270 μL	Unidirectional (25 µL/h)	No mechanical stimulation	No specific control	[58]
PDMS	Soft lithography	Single microchannel with a culture chamber	Not stated	Unidirectional (45 µL/h)	No mechanical stimulation	No specific control	[59]
PC	Controlled milling and laser-cut	Three microchannels separated by two semi-permeable membranes	4 mm W × 200 mm L × 0.5 mm H	Unidirectional (16 µL/min)	No mechanical stimulation	Luminal anoxic medium (anaerobic chamber)	[60]
PDMS	3D-printing-assisted soft lithography	Single channel	Not stated	Unidirectional (60 µL/h)	No mechanical stimulation	No specific control	[61]
PDMS	Soft lithography	Two micro-channels separated by a PC membrane	Not stated	Unidirectional (100 µL/h)	No mechanical stimulation	Luminal anoxic medium (anaerobic chamber)	[62]
PDMS	Micromilling- assisted soft lithography	Three microchannel layers separated by polycarbonic acid membranes	Not stated	Unidirectional (60 µL/h)	No mechanical stimulation	Luminal anoxic medium (L-cysteine and nitrogen perfusion)	[63]
PDMS	Not stated	Not stated	Not stated	Unidirectional (120 µL/h)	No mechanical stimulation	No specific control	[64]
PDMS	Soft lithography	Two channels separated by shield-shaped micropillars	700 μm W, 0.65 cm L and 150 μm H	Unidirectional (Not stated)	Cyclic strain (15%, 0.2 Hz)	Co-culture in anaerobic incubator	[30]
PS	Not stated	Three parallel channels	Not stated	Bidirectional (Not stated)	No mechanical stimulation	No specific control	[65]
PS	Not stated	Three parallel channels	Not stated	Bidirectional (Not stated)	No mechanical stimulation	Co-culture in anaerobic incubator	[66]
PC	Manual milling	Two channels separated by a PC membrane	Not stated	Unidirectional (Not stated)	No mechanical stimulation	No specific control	[67]
PDMS	3D printing-based fabrication protocol	Two channels separated by polyester membrane	Upper: 750 μm H × 11 mm L; Lower: 200 μm H × 11 mm L	Unidirectional (60 μL/h)	No mechanical stimulation	No specific control	[33]
Not stated	Not stated	Not stated	Not stated	Not stated	No mechanical stimulation	No specific control	[31]
PDMS	Soft lithography	Two layers (top and bottom)	Not stated	Unidirectional (45 μL/h)	Cyclic strain (parameters not stated)	No specific control	[68]
PDMS	Soft lithography	Two microchannels separated by a PDMS membrane	Not stated	Unidirectional (30 μL/h)	Cyclic strain (No stretch during infection)	Luminal anoxic medium (anaerobic chamber)	[69]
PDMS	Soft lithography	Two microchannels separated by a PDMS membrane	Not stated	Unidirectional (120 μL/h)	Cyclic strain (10%, 0.15 Hz)	No specific control	[36]
PC	Controlled milling and laser-cut	Four parallel microfluidic channels	Not stated	Unidirectional (16 µL/min)	No mechanical stimulation	Luminal anoxic medium (anaerobic chamber)	[70]
PDMS	Soft lithography	Five channels	Not stated	Unidirectional (100 µL/h)	No mechanical stimulation	No specific control	[71]
PDMS	Soft lithography	Single channel	1.5 mm W × 10 mm L ×2 mm H	Unidirectional (40 µL/h)	No mechanical stimulation	No specific control	[32]

Abbreviations: H: height; L: length; PC: polycarbonate; PDMS: polydimethylsiloxane; PS: polystyrene W: width.

From a design perspective, the basic structure of a GoC is a device consisting of a single microchannel in which cells are cultured along its length (*n* = 4). It is connected to a peristaltic pump to allow medium flow [61,68]. Owing to its simplicity, this configuration may be regarded as analogous to a conventional cell culture insert. More complex GoCs include two microchannels (*n* = 32), three microchannels *(n* = 7) or multiple microchannels (*n* = 2). The microchannels can either be separated by a porous membrane [42,43,69] or have no physical division along their channels [65,66]. This reflects the diversity of existing GoC *in vitro* architectures. For microbiome experiments, these microchannel architectures allow continuous and precisely controlled flow and the establishment of defined chemical and oxygen gradients, while insert configurations are restricted to limited superficial flow and poorly defined gradients. In addition, several channels can represent different compartments of the gut, such as the intestinal lumen and the vascular side, allowing the generation of separate but interacting culture environments. For this reason, multi-channel chips are often preferred over single-microchannel chips.

#### 3.2.2. Perfusion and Stretch

According to the characteristics of the flow along the chips, most GoC devices used in microbiome studies have unidirectional flow (*n* = 44), which is mainly achieved through the use of pumps. Only a minority of them (*n* = 2) have bidirectional flow due to the use of gravity-driven flow generated by a customized rocker platform. In both systems, a similar degree of intestinal cell differentiation is achieved [40,65], including the formation of three-dimensional structures resembling intestinal villi, enhanced expression of tight junctions and mucus production. Unidirectional flow is the most employed configuration in GoC platforms and can mimic the continuous luminal transport of nutrients, metabolites, and microbial products along the gastrointestinal tract while exposing epithelial cells to physiologically relevant shear stress. In contrast, bidirectional or oscillatory flow may better reproduce aspects of the local fluid movements associated with intestinal motility and luminal mixing and metabolite exchange. Regarding microbiome studies, a potential limitation of bidirectional GoC systems could be the repeated exposure of cells to accumulated microorganisms, together with their metabolites, secreted factors, cellular debris, and damage-associated molecular patterns (DAMPs). However, the impact of such recirculation has not been systematically evaluated, and there are no direct comparative studies evaluating the impact of the different flow patterns in GoC systems, making it difficult to determine which configuration is most appropriate for specific physiological applications.

Only selected GoC studies (*n* = 19) have incorporated stretch in their development, which emulates the peristaltic processes of the intestinal wall, and considerable heterogeneity exists across the published studies. Experimental conditions vary substantially, with cyclic strain values typically ranging from 5% to 15%, while the frequencies applied are most commonly between 0.15 and 0.20 Hz. The limited number of studies directly comparing these parameters under standardized conditions currently hampers definitive conclusions regarding their individual contributions to the observed biological responses. Although gut peristalsis simulation is not required for an efficient formation of villi-like structures inside the chips, it may play an important role in host–microbial interactions when a microbiome is inoculated in the GoC by promoting selective adhesion or propagation of the microorganisms. In this context, infection and propagation in Caco-2 GoC were enhanced by peristaltic stretching without affecting its adhesion properties [35]. By contrast, *Escherichia coli* colonization was reduced when GoC experienced mechanical cyclic deformations [40]. These results point to different mechanisms involved in host–microbial interactions in the GoC. However, more studies are required to elucidate the role of stretch in complex microbiome studies and would constitute a field for further research.

#### 3.2.3. Oxygen Environment

Few standardized methods exist for reproducing, in GoC devices, the physiological oxygen gradients found in the gut. Typically, hypoxia develops spontaneously as oxygen diffusion is restricted by the chip architecture and further depleted by cellular metabolism. Therefore, most GoC systems used in microbiome studies (*n* = 30) lack mechanisms for generating hypoxic conditions, which represents a limitation of these models. By contrast, some platforms have enabled a straightforward strategy to generate an oxygen gradient by perfusing pre-deoxygenated medium exclusively through the apical channel of the device (*n* = 18). Using this approach, physiologically relevant hypoxic conditions have successfully been established to support the co-culture of obligate anaerobic probiotics [43,60]. The deoxygenated medium can be achieved by equivalent strategies such as the addition of L-cysteine [54,63] or the use of anaerobic jars [56]. In addition, placing the chip in a hypoxic incubator (*n* = 2) represents another possible alternative [66].

#### 3.2.4. Cell Sources

First, functional GoCs were developed using well-characterized epithelial intestinal cell lines due to their high proliferation rates and simple culture requirements. Caco-2, HT-29 or a co-culture with both lineages is suitable for screening purposes [12,50]. For Caco-2 cells, both parental cell line Caco-2 (HTB-37) and clones BBE, TC-7 clones from the American Type Culture Collection (ATCC), as well as Caco-2 (ACC169) from the German Collection of Microorganisms and Cell Cultures (DMSZ), have been validated as proof-of-concept of this technology. Of note, several laboratories have reported that Caco-2 cells differentiate into a polarized columnar epithelium that contains cells with markers of absorptive, mucus-secretory, enteroendocrine, and Paneth cell populations [12,44], pointing out its relevance as a model despite its tumorigenic origin. The GoC may include HT-29, HT-29-MTX or HT-29-MTX-E12 cell lines to increase the population fraction of mucus-producing cells to better mimic the physiological mucus barrier.

To generate more physiologically relevant models, GoC devices can be constructed using primary enterocytes isolated from donor biopsies or intestinal explants, which better replicate the *in vivo* epithelial architecture of specific intestinal regions, such as the duodenum or the colon [21]. Such devices facilitate the characterization of spatially resolved host–microbiome interactions, providing a controlled environment to dissect region-specific physiological responses within the intestinal epithelium. However, these approaches typically require ethics committee approval and depend on a continuous supply of clinical samples. In order to overcome this limitation, stem-cell-derived models and organoids have emerged as an attractive alternative cell source for GoC systems to carry out microbiome studies. Three-dimensional organoid cultures can be generated from either adult intestinal stem cells isolated from biopsies or induced pluripotent stem cells [46,47]. After organoid dissociation, the resulting cells can be seeded into microfluidic devices, where they undergo spontaneous differentiation and give rise to the major lineages present in the gut, including absorptive enterocytes, enterochromaffin cells, goblet cells, and Paneth cells.

The selection of the most appropriate cell source requires balancing physiological relevance with experimental reproducibility and ultimately depends on the specific scientific question being addressed. Although immortalized cell lines provide a well-established, reproducible, and standardized experimental platform, they do not fully recapitulate the characteristics of the native human intestinal epithelium, partly due to their tumor-derived origin. In contrast, primary cells and patient-derived organoids are generally considered more physiologically relevant, as they more closely resemble the cellular diversity and functional properties of native intestinal tissue. However, their use is associated with greater variability in experimental outcomes due to donor-to-donor heterogeneity and differences in culture establishment and maintenance, posing challenges for standardization and reproducibility. Consequently, each cell source offers distinct advantages and limitations, and its suitability should be evaluated in the context of the specific objectives of the study.

As the gut epithelium is supported in its function by a collaborative and intricate vascular network that forms a supplementary barrier, several GoCs used in microbiome studies have been developed as a co-culture of epithelial and endothelial cells, which may enhance the barrier function and mucus production [43]. Human umbilical vein endothelial cells (HUVECs) or human intestinal microvascular endothelial cells (HIMECs) are the most frequent cell sources for endothelial cells [46,55]. This approach will enable researchers to assess the endothelial function in the context of microbiome co-cultures, providing insights into how microbial communities may influence vascular behavior.

In addition to endothelial cells, certain microbiome studies with GoCs include immune cells, which are essential for the maintenance of intestinal homeostasis. However, integrating immune cells in the GoC may be challenging, and two complementary approaches have been proposed. First, immune cells can be perfused into the endothelial channel to mimic lymphocyte infiltration from the bloodstream [19,39,40,41,58]. In the second strategy, immune cells are seeded in the microchannel together with epithelial cells or endothelial cells, which would simulate resident macrophages present in the gut [44,53]. Innate immunity can be modelled with either monocytic cell lines such as THP-1, U937 and dendritic cell line MUTZ-3 [18] or primary cells [39,53,66], including peripheral blood mononuclear cells (PBMCs), monocyte-derived macrophages purified from PBMCs or neutrophils. By contrast, adaptive immunity has scarcely been investigated in microbiome studies using GoC [39] and therefore could form the basis for future research.

### 3.3. Host–Microbiome Studies in GoC Devices

Microbiome studies (*n* = 36) represent the most frequent research focus among the GoC models reviewed and include investigations of host–microbiome interactions with varying degrees of complexity (Table 2). As shown in Table 2, only seven studies incorporate faecal-derived microbiota in their experimental design [21,29,30,43,47,49,62] while ten studies [18,28,32,40,44,48,49,55,59,71] included both microbiome studies with non- and pathogenic microorganisms. Pathogen mechanistic studies (*n* = 13) are summarized in Table 3. One publication is included in both the microbiome and pathogen mechanistic studies [33]. Markedly, only one of the publications included a postbiotic intervention among the experimental conditions assessed [65].

**Table 2:** Detailed overview of microbiome and nutritional intervention studies using gut-on-a-chip (GoC) models.

Cell Types			Microbiota		Co-Culture (Days)	Application	Major Biological Outcomes	Reference
Intestinal Cells	Immune Cells	Endothelial Cells						
Caco-2 (BBE)	NA	NA	*Lacticaseibacillus rhamnosus* GG (ATCC 53103)	NA	7	Development of the first GoC model for co-culture with a probiotic strain	Improved barrier function: TEER	[12]
Caco-2 (HTB-37)	NA	NA	*L. rhamnosus* GG (LMG 18243) Microbiome obtained from a fermented faecal sample in SHIME^®^ reactor	NA	2	Evaluation of the anti-inflammatory activity	Decrease in IL-8 secretion	[38]
Caco2 (ACC169) CCD-18Co (CRL-1459)	Primary CD4+ T cells	NA	*L. rhamnosus* GG (ATCC 53103) *Bacteroides caccae* (DSMZ 19024)	NA	1	Analysis of transcriptional, metabolic and immunological host response	Transcriptomic profile comparable to clinical samples Accumulation of GABA Decrease in IL-8 and CCL20 secretion	[39]
Caco-2 (BBE)	Peripheral blood mononuclear cells (PBMCs)	Capillary cells Lymphatic microvascular cells	VSL#3 probiotic blend *Escherichia coli* (DH5-GFP)	*E. coli* O124:NM (ATCC 43893)	4	Model of small intestinal bacterial overgrowth (SIBO) and evaluation of the barrier integrity	Improved barrier function: TEER	[40]
Caco-2 (BBE)	PBMCs	NA	VSL#3 *E. coli* (D21f21-GFP)	NA	10	Evaluation of the anti-inflammatory activity and barrier protection	Improved barrier function: TEER Decrease in IL-1β, IL-6, and TNF-α secretion	[41]
Caco-2 (ACC169)	NA	NA	*L. rhamnosus* GG (ATCC 53103)	NA	1	Analysis of diet-microbiome-host interactions on hallmarks of cancer	Downregulation of pro-carcinogenic and drug resistance genes	[42]
Caco-2 (BBE) Intestinal organoids	NA	Human intestinal microvascular cells (HIMECs)	*Bacteroides fragilis* (NTCT 9343) Colon/caecum samples from mice colonized with human microbiota faecal samples from infants	NA	5	Analysis of the diversity of microbiota co-cultured under physiologically relevant oxygen gradients	Improved barrier function: P_app_ Sustained physiologically relevant microbial diversity	[43]
Caco-2	Macrophages differentiated from PBMCs	Human umbilical cord vein cells (HUVECs)	*L. rhamnosus* (ATCC 7469)	*Candida albicans* (SC5314)	1	Model of immunocompetent intestine-on-chip and colonization by *C. albicans*	Improved barrier function: P_app_ and expression of E-cad and Zo-1 Growth and translocation inhibition of *C. albicans*	[44]
Caco-2 (BEE)	NA	NA	*Bifidobacterium adolescentis* (DSM 20083) *Eubacterium hallii* (DSM 17630)	NA	3	Model of GoC for co-culture with obligate anaerobic strains	Improved barrier function: TEER	[45]
Caco-2 (HTB-37)	NA	HUVECs	*Lacticaseibacillus casei* L5 BGB	*E. coli* (ATCC 11775)	4	Evaluation of the anti-inflammatory activity and barrier protection	Improved barrier function: TEER and P_app_ and occludin expression Decrease in IL-1β, IL-6, IL-8 and TNF-α secretion	[18]
Caco-2 (BBE) Intestinal organoids	NA	NA	Faecal sample from a healthy donor	NA	2	Patient-specific models of human chronic gastrointestinal disorders	Maintained barrier function: TEER	[47]
Caco-2 (BBE)	NA	NA	*Bifidobacterium breve* (ATCC 15700)	*E. coli* (Hu734)	4	Assessment of the epithelial barrier morphology upon a pathogen challenge	Preservation of the epithelial cell layer thickness	[48]
Mouse colon organoids	NA	NA	*Enterococcus faecium* Human faecal samples Mouse colon samples	*Salmonella enterica* serovar Typhimurium (SL1344)	2	Assessment of bacterial strains that modulate host response to pathogens	Decrease in CXCL1 secretion Cell viability protection Improved barrier function (Zo-1)	[49]
Caco-2 (BBE) HT-29 MTX-E12	NA	Human microvascular cells (HMVECs)	*E. coli* Nissle 1917 (SYNB1618)	NA	0.5	Preclinical model of phenylketonuria disease and evaluation of a live bacterial therapeutic	Reduction of phenylalanine concentration	[50]
Caco-2 Primary intestinal subepithelial myofibroblasts	PBMCs	NA	*L. rhamnosus* GG (ATCC 53103) *Bifidobacterium longum* subsp. *infantis* (ATCC 15697)	NA	0.7	Evaluation of the anti-inflammatory activity and barrier protection	Improved barrier function: TEER, Zo-1 expression Decrease in IL-8, IL1-α, IL-2, IFN-γ secretion	[51]
Caco-2 (HTB-37)	NA	HUVECs	*Lacticaseibacillus plantarum* (HY7715) *Bifidobacterium animalis* subsp. *lactis* (HY800) *L. plantarum* (ATCC14917)	NA	5	Evaluation of the adhesion, anti-inflammatory and activity and barrier protection	Improved barrier function: TEER, Zo-1 expression and villus height	[52]
Caco-2	NA	NA	*L. plantarum*	*E. coli* BL21 (DE3)	1	Evaluation of the barrier protection	Improved barrier function: P_app_, Zo-1 and occludin expression	[28]
Caco-2 (BBE)	NA	NA	*L. rhamnosus* GG (ATCC 53103) VSL#3	NA	3	Evaluation of the anti-inflammatory activity and barrier protection	Improved barrier function: TEER, Zo-1 and occludin expression Decrease in IL-1β, TNF-α, and IL-8 secretion and p65, pSTAT3 and MYD88 expression	[54]
Caco-2	PBMCs	HUVECs	*L. rhamnosus* GG (ATCC 53103)	*E. coli* with extended Beta-lactamase	1	Evaluation of the anti-inflammatory activity and barrier protection of probiotics and antibiotics	Improved barrier function: P_app_ Decrease in IL-6 secretion	[55]
Neonatal ileal enteroids	NA	HIMECs	Microbiota sample from a patient with enterocolitis	NA	3	Model of neonatal necrotizing enterocolitis: transcriptional and inflammatory response	Decreased in barrier function: P_app_, Zo-1 expression Inflammatory gene expression: IL-1β and IL-8	[21]
Caco-2	NA	NA	*Bifidobacterium bifidum* (BNCC 186493)	NA	2	Evaluation of the anti-inflammatory activity and barrier protection	Improved barrier function: TEER and P_app_ Zo-1 expression Decrease in IL-1β, IL-6, IL-8 and TNF-α secretion	[56]
Caco-2 (BEE) HT-29 MTX-E12	NA	HMVECs	*E. coli* Nissle 1917 (SYN)	NA	1	Preclinical model of cortisol metabolization	Metabolization of L-tryptophan Improved barrier function: P_app_	[57]
Human colon tissue explants	NA	NA	Human faecal samples	*NA*	1	Evaluation of the anti-inflammatory activity and barrier protection	Improved barrier function: P_app_ Decrease in IL-1β, IL-6, IL-8 and TNF-α secretion	[29]
Caco-2	NA	NA	*E. coli* (ATCC 25922) *B. fragilis* (ATCC 25285) *L. rhamnosus* GG (ATCC 53103)	*B. fragilis* (ATCC 43858) *B. fragilis* (ATCC 43859)	6	Model of pro-tumorigenic signaling activation and evaluation of probiotics	Inhibition of the adhesion Improved barrier function: Zo-1	[59]
Caco-2 (ACC169)	THP-1	HUVECs	*E. coli* MG1655 (DMSZ 18039)	NA	0.25	Model of drug metabolism along the gut–liver axis	Transformation of the inactive metabolite SN-38G into its toxic metabolite SN-38 by *E. coli*	[60]
Caco-2 (CL-0050)	NA	NA	Faecal samples of patients with depression and healthy volunteers	NA	2	Preclinical model of GoC associated with depression: assessment of inflammation, barrier function and 5-HT content	Decrease in 5-HT secretion and Zo-1 expression and increase in IL-8 by depression patient samples	[62]
Caco-2 (HTB-37) HT-29 (HTB-38)	NA	NA	*B. longum* and *B. longum* subsp. *infantis* (BL#1-1; BL#1-3; BL#1-4; BL#1-5; BL#1-6; BL#1-7; BL#1-9 BL#1-10; BL#3-4, BL#3-12, BL#3-13; BL#3-14)	NA	3	Evaluation of the anti-inflammatory activity and barrier protection	Improved barrier function: TEER and P_app_ Decrease in IL-1β and TNF-α secretion	[63]
Caco-2 HT-29-MTX HMEC-1 Intestinal organoids	NA	NA	Human faecal samples	NA	1	Evaluation of the microbiota of melanoma patients who are responsive or not to immunotherapy	Expression of pro-inflammatory genes in non-responsive patients Improved barrier function: Papp and Zo-1 in responsive patients	[30]
Caco-2 (TC-7)	PBMCs	NA	Conditioned medium: *L. rhamnosus* GG (DSM 33156), *B. longum* subsp. *infantis* (DSM 33361) *B. longum* subsp. *infantis* (ATCC 15697) *B. longum* subsp. *infantis* (Bi-26) *B. longum* subsp. *infantis* (EVC001)	NA	2	Evaluation of the anti-inflammatory activity and barrier protection	Improved barrier function: TEER and P_app_ Decrease in pro-inflammatory cytokine secretion	[65]
Caco-2 (TC-7)	PBMCs	NA	*Bacteroides thetaiotaomicron* (DSM 72079) *Akkermansia muciniphila* (DSM 22959) *Christensella minuta* (DSM 22607) *Roseburia intestinalis* (DSM 14610) *Clostridium scindens* (DSM 5676) *Adlercreutzia equolifaciens* (DSM 19450)	NA	2	Evaluation of the anti-inflammatory activity and barrier protection	Improved barrier function: TEER and Zo-1 Decrease in pro-inflammatory cytokine secretion	[66]
Caco-2 HT-29-MTX-E12	NA	NA	*L. rhamnosus* GG	NA	5	Model of biofilm formation	Improved barrier function: P_app_ and mucus production	[67]
Caco-2 (TC-7)	NA	NA	*L. plantarum* NCIMB8826	NA	1	Model of adhesion	Number of bacteria adhered	[33]
Caco-2	NA	NA	*E. coli* Nissle 1917 *L. rhamnosus GG* (ATCC 53103)	NA	1	Model of gut motility	Adhesion of the bacteria and villi height	[68]
Caco-2 (ACC169)	PBMCs	NA	17-strain *Clostridia* blend	NA	1	Analysis of transcriptional, metabolic and immunological host response	Bacterial relative abundances and metabolite production Secretion of IFN-*γ*, IL-2, IL-6 and TNF-*a*.	[70]
Caco-2 (CL-0050) HCT-116 (CL-0096)	NA	HUVECs	*E. coli* BW25113 and variants	*S. enterica*	1	Model of biofilm formation and pathogen colonization	Adhesion of bacteria Analysis of the metabolism	[71]
Caco-2 (cat#1102HUM -NIFDC00087) HT-29 (cat#3101HUM SCSP5032)	NA	NA	*E. coli* Nissle *1917*	*S. enterica* serovar Typhimurium	1	Evaluation of the adhesion and barrier protection	Improved barrier function: Zo-1 expression Proteomic analysis	[32]

Abbreviations: NA: Not applicable.

**Table 3:** Detailed overview of pathogen mechanistic insight studies using gut-on-a-chip (GoC) devices.

Cell Types			Microbiota	Co-Culture (Days)	Application	Major Biological Outcomes	Reference
Intestinal Cells	Immune Cells	Endothelial Cells					
Caco-2 (BBE)	NA	NA	Coxsackievirus B1 (ATCC VR-28)	2	Infection and inflammatory response	Virus release polarized towards the epithelial lumen Secretion of IL-8 and IP-10	[34]
Caco-2 (TC-7)	NA	NA	*Shigella flexneri* 5a (M90T), *mxiD* mutant and *icsA* mutant	0.6	Infection and invasion	Peristaltic motion enhances *Shigella* infection invasion	[35]
Colonic organoids	NA	HIMECs	*E. coli* (EDL931)	2	Infection and inflammatory response	Secretion of inflammatory cytokines in the GoC similar to *in vivo* infection	[46]
Caco-2 HT-29 (CL-0118)	PBMCs	HUVECs	*Severe acute respiratory syndrome-related* *coronavirus* (SARS-CoV-2) strain 107	3 h	Infection, epithelial barrier and transcriptional analysis	Decrease in barrier function: mucin, VE-cadherin Transcriptomic analysis: induction of cytokines	[19]
HT-29	NA	NA	*E. coli, T4 phages*	1	Potential tripartite evolutionary interactions between phages, bacterial host and gut mucosa	Phage mutation analysis	[27]
Caco-2	Primary macrophages	HUVECs	*E. coli* (11775)	2	Infection and inflammatory response	Decrease in barrier function: P_app_; secretion of cytokines IL-8, IL-6, TNF-α	[53]
Caco-2	PBMCs	HUVECs	*C. albicans* (SC5314) *C. albicans* (110.12)	1	Infection and inflammatory response	Decrease in barrier function: P_app_ and secretion of IL-1β, IL-8, IL-6, TNF-α cytokines	[58]
Caco-2 (HTB-37) LS 174T (CL-138)	NA	NA	*Pseudomonas aerugionosa* CN573 *E. coli* (ST131) Phages: PNM, HP3, ES17	3	Infection	Bacterial loads over time	[61]
HT-29 (HTB-38)	NA	NA	*E. coli* (SH232) Phage øPNJ-6	1	Infection	Phage adhesion to mucus Mucus production	[64]
Caco-2 (TC-7)	NA	NA	*S. flexneri (M90T-GFP)*	1	Adhesion	Bacterial loads	[33]
Caco-2 (CRL2102) HT-29 (HTB-38)	NA	NA	*C. albicans* SC5314, 3 oak tree, 1 sugar-adapted isolates	1	Infection (colonization, invasion and translocation)	Yeast loads Cellular damage (LDH)	[31]
Caco-2 (TC-7) HT-29-MTX	NA	NA	*Clostridioides difficile* (CD1611) *Clostridioides difficile* (CNRS_CD129) *Clostridioides difficile* (CNRS_CD345)	2	Infection	Cytotoxicity Bacterial loads	[69]
Caco-2 (HTB-37) HT-29 (HTB-38)	NA	HIMECs	*S. enterica* serovar Typhimurium (SL1344)	1	Infection	Bacterial loads	[36]

Abbreviations: NA: Not applicable.

#### 3.3.1. Microbiome and Nutritional Studies

GoC studies incorporating microbial components have been conducted with varying degrees of complexity, ranging from models using a single gut microbe, such as a probiotic strain or commensal microorganism, to those employing commercial multispecies probiotics or complex communities derived from faecal samples. Early work by Kim et al. [12] using a GoC platform demonstrated that co-culture of Caco-2 cells with the probiotic *Lacticaseibacillus rhamnosus* GG enhanced epithelial barrier function, consistent with observations reported in human studies. Subsequent research expanded on these findings by evaluating the effects of probiotic strains using diverse commercial and custom-built GoC systems. For example, De Gregorio et al. [51], Liu et al. [56], Min et al. [54], Wu et al. [63] and Dash et al. [67] reported that *L. rhamnosus* GG, *Bifidobacterium bifidum* BNCC186493, and *Bifidobacterium longum* BL#3-14 exerted protective effects against inflammatory stimuli by preserving villus-like structures, regulation of tight junction proteins or by a homeostatic effect on cytokines and epithelial tightness. Similarly, Jeon et al. [52] observed that the co-culture of a damaged epithelial layer with the probiotic *L. plantarum* HY7715 resulted in a substantial recovery of barrier function. Moreover, Liu et al. [56] demonstrated that probiotic *Bifidobacterium bifidum* BNCC 186493 both prevented and reversed epithelial barrier disruption induced by tumor necrosis factor-alpha (TNF-α) and lipopolysaccharide (LPS).

In contrast to studies evaluating individual probiotic strains, Kim et al. [40], Shin and Kim [41] and Min et al. [54] investigated the commercial multispecies probiotic formulation VSL#3. In these studies, VSL#3 significantly improved epithelial barrier integrity, as indicated by increased transepithelial electrical resistance (TEER).

A more advanced GoC model for evaluating nutritional interventions was presented by Greenhalgh et al. [42], who examined the combined effects of a prebiotic medium and *L. rhamnosus* GG. Compared to either component alone, the symbiotic regimen downregulated genes associated with procardiogenic pathways and drug resistance, and reduced levels of the oncometabolite lactate. Their findings highlighted the potential of GoC systems for the rational design and evaluation of specific symbiotic formulations.

In a complementary study, Marzorati et al. [38] coupled effluents from the SHIME^®^ dynamic gastrointestinal model with a GoC device to investigate how specific functional ingredients modulate both luminal microbial communities and host epithelial responses. Fermentation of a commercial yeast product altered microbiota composition within the gastrointestinal simulator, while the corresponding effluents reduced Interleukin (IL)-8 secretion by Caco-2 cells over 24–48 h, supporting the anti-inflammatory properties of the *Saccharomyces cerevisiae* fermentation product Epicor^®^. However, in this case, a nanoporous membrane bearing an artificial mucus layer was required to separate microorganisms from human cells.

In the healthy gastrointestinal tract, commensal microbiota play an essential role in preventing colonization by pathogenic organisms, thereby reducing infection risk and inflammation. Therefore, the ability of commensal gut microbiota to limit pathogen colonization has also been explored using GoC platforms. Reflecting this protective function, numerous studies have employed GoC platforms to examine commensal microbiota–pathogen interactions [18,44,48,49,55,59,71]. In these assays, bacteria such as *Lacticaseibacillus casei* and *Bifidobacterium breve* or the probiotic *L. rhamnosus* GG mitigated epithelial damage and attenuated inflammatory responses in the GoC induced by either *E. coli* or enterotoxigenic *Bacteroides fragilis*.

Several studies have also employed GoC platforms to evaluate synthetic live bacterial therapeutics. Nelson et al. [50] demonstrated the utility of these systems for assessing the function and efficacy of genetically engineered microbial strains under physiologically relevant conditions. In their more recent work, Nelson et al. [57] investigated a bioengineered *E. coli* Nissle 1917 strain designed to sense cortisol at physiological concentrations and activate a genetic circuit encoding tryptophan decarboxylase, thereby converting tryptophan to tryptamine. The GoC enabled assessment of tryptophan and tryptamine metabolism and apparent permeability across the epithelial–vascular interface. In an earlier study, Nelson et al. [50] showed that a similar GoC device could recapitulate *in vivo* activity observed in a clinical trial of a live biotherapeutic intended for the management of phenylketonuria, a metabolic disorder caused by impaired phenylalanine hydroxylase function. Collectively, these findings support the application of GoC systems as preclinical efficacy platforms for evaluating engineered microbial therapeutics.

The oxygen sensitivity of strict or obligate anaerobic bacteria has historically posed major challenges for *in vitro* models aimed at studying host–microbe interactions, particularly when co-culturing these microbes with oxygen-dependent intestinal epithelial cells. Several research groups [39,43,45,47] therefore focused on determining whether GoC platforms could sustain dynamic, direct interactions between living, mucus-producing human intestinal epithelium and anaerobic microorganisms. Shah et al. [39] and Shin et al. [45] successfully cultured defined anaerobic species, including *Bacteroides caccae*, *Bifidobacterium adolescentis*, and *Eubacterium hallii*, in direct contact with human epithelial cells. In a complementary work, Jalili-Firoozinezhad et al. [43] and Shin et al. [47] demonstrated that GoC systems could also support more complex microbial communities derived from faecal samples, comprising both aerobic and anaerobic commensals in direct contact with human intestinal epithelial cells with oxygen concentrations of less than 0.3% in the epithelial channel. However, although these platforms support the survival and growth of increasingly complex microbial consortia, the extent to which the cultured communities remain representative of the original donor microbiota remains an important consideration. Culture conditions, including oxygen gradients, nutrient availability, flow dynamics, and host-derived factors, may selectively enrich or deplete specific taxa, leading to changes in microbial diversity and community structure that could influence experimental outcomes and their biological interpretation. More recently, De Rudder et al. [70] advanced this field by culturing a probiotic mixture comprised of 17 strictly anaerobic clostridial strains in a GoC composed of epithelial cells and PBMCs from different donors. The model successfully sustained metabolically active microbiota while maintaining host cell viability and enabled the detection of increased cytokine production, indicative of active immune sensing by PBMCs. These findings highlight the potential of such immunocompetent GoC platforms as personalized tools for investigating microbiota-driven immune responses and for preclinical screening applications. Collectively, these studies showed that GoC platforms can sustain strict anaerobes while enabling investigation of key physiological interactions with host tissues, including processes relevant to intestinal metabolism, epithelial homeostasis, and immune regulation.

One of the most promising frontiers in physiological systems is the use of GoC platforms to model patient-specific pathophysiology through the integration of human gut microbiota and intestinal tissues. This approach enables researchers to recapitulate complex host–microbe interactions under controlled conditions, advancing both mechanistic research and personalized therapeutic development. A compelling example of this strategy is the work by Wang et al. [62], who developed a microfluidic “depression-GoC” model containing gut microbiota from patients with major depressive disorder. Their system enabled stable co-culture of human intestinal epithelial cells with anaerobic gut microbiota and accurately reproduced key physiological hallmarks observed in depressed individuals, including reduced intestinal barrier function, chronic low-grade inflammation, and decreased serotonin (5-HT) levels. These findings highlight the feasibility of using patient-derived microbiota to study bidirectional gut–brain axis mechanisms and identify novel biomarkers for psychiatric disorders. Nevertheless, patient-derived microbiota models are subject to donor-to-donor variability arising from factors such as disease stage, medication use, diet, lifestyle, and baseline microbial composition, which may influence the phenotypes observed. Although such variability enhances the physiological relevance of these platforms, it may also complicate reproducibility and limit direct comparison between studies.

The physiological relevance of GoC platforms can be further enhanced by integrating human intestinal organoids derived from patient biopsies in combination with patient faecal-derived microbiota. Such organoid-based systems retain patient-specific genetic, epigenetic and disease-related features, offering a more faithful representation of disease states than immortalized cell lines. Jalili-Firoozinezhad et al. [43] demonstrated that GoC systems can support the co-culture of complex fresh gut microbiome derived from faecal samples from four infants, in direct contact with the human intestinal epithelial cells obtained from organoids derived from normal regions of surgical biopsy of human ileum and its overlying mucus layer for multiple days. In a complementary work, Shin et al. [47] optimized a methodology for robust attachment and long-term culture of biopsy-derived human intestinal organoid epithelium within PDMS-based GoC devices. Their platform enabled the generation of patient-specific chips using organoids derived from individuals with Crohn’s disease, ulcerative colitis, and colorectal cancer. Under microphysiological flow and mechanical deformation, these patient-derived epithelial layers exhibited disease-specific architectures and functional phenotypes. Co-culture with human faecal microbiota in an anoxic–oxic interface further demonstrated stable host–microbe interactions without loss of barrier integrity, underscoring the potential of these hybrid systems for modeling complex inflammatory gut disorders. However, outcomes generated using biopsy-derived tissues may also be influenced by variables, including biopsy location, disease severity, prior treatments, sample handling procedures, and culture conditions. Consequently, careful consideration of these factors is required when comparing results across donors or between independent studies.

Extending the patient-specific approach to neonatal conditions, Lanik et al. [21] created a microfluidic necrotizing enterocolitis (NEC)-on-a-chip model using intestinal enteroids derived from premature infants and microbiota isolated from an infant who succumbed to NEC. This system successfully recapitulated hallmark NEC features, including heightened pro-inflammatory cytokine expression, impaired epithelial proliferation, and compromised barrier integrity. As with other patient-derived platforms, the translational value of this approach stems from its ability to capture clinically relevant heterogeneity, although such heterogeneity may also contribute to experimental variability and challenges in standardization. By reproducing the dysbiosis-driven inflammatory environment characteristic of NEC, this model provides a tool for mechanistic studies and supports the development of urgently needed therapeutics for this devastating condition.

Collectively, these studies demonstrate that GoC platforms incorporating both patient-derived microbiota and tissues provide valuable insight into individual-specific disease mechanisms. Compared with traditional *in vitro* or animal models, these systems might:

- Capture host genetic background and epithelial functionality through patient-derived organoids.
- Maintain physiological host–microbe interactions under controlled microenvironmental conditions.
- Enable modeling of diseases previously difficult to study due to limited human tissue availability (e.g., NEC).
- Provide a tractable platform for high-resolution mechanistic studies and preclinical testing of targeted therapeutics.
- As these technologies advance, they are likely to play a central role in supporting the development of probiotics, postbiotics and nutraceutical interventions in their preclinical stage.

#### 3.3.2. Pathogen Mechanistic Insights

A minority of the research employing GoC platforms has focused on modelling infectious processes in the human intestine (Table 3). These systems enable controlled recreation of host–pathogen interactions, including epithelial responses, barrier dysfunction, and immune activation.

Early GoC infection studies demonstrated the platforms’ ability to distinguish between commensal and pathogenic bacterial activity. Kim et al. [40] compared the effects of non-pathogenic *E. coli* with enteroinvasive *E. coli* (EIEC; O124:NM). While commensal *E. coli* did not disrupt epithelial function, EIEC infection via the apical channel resulted in complete barrier breakdown and destruction of villus-like structures within 24–36 h, mirroring severe diarrheal disease in humans.

Subsequent studies expanded the applicability of these models to a disease-specific context. For instance, Jing et al. [53] developed an inflammatory bowel disease model induced by *E. coli* 11775 and demonstrated that chitosan oligosaccharides can protect the intestinal epithelial barrier and vascular endothelial barrier by inhibiting adhesion and invasion. Furthermore, Tovaglieri et al. [46] use a GoC platform to model human infection with enterohemorrhagic *E. coli* (EHEC; O157:H7). By integrating metabolites generated from murine or human microbiota, the study revealed that human-derived metabolites significantly increased EHEC motility, epithelial injury, and overall pathogenicity. Specific human microbiome-derived metabolites such as 4-methyl benzoic acid, 3,4-dimethylbenzoic acid, hexanoic acid and heptanoic acid were identified as key mediators, highlighting the microbiome’s modulatory influence on virulence.

Similarly, Zhao et al. [55] used a GoC to study infection by extended-spectrum β-lactamase-producing *E. coli*. The infection induced microvilli damage, reduced mucus secretion, extensive cell death, and increased secretion of IL-8, IL-6, IL-1β, and TNF-α. These results are consistent with previous *in vivo* findings [6], further validating the physiological relevance of GoC infection models.

Additional work has examined *Shigella flexneri* and *Salmonella enterica* serovar Typhimurium infections [35,36,49]. Across these studies, peristalsis-like mechanical stimulation accelerated epithelial invasion and tissue destruction, with *S. flexneri* localizing among villus-like structures. In *Salmonella* models, epithelial detachment, reduced tight-junction expression, and increased chemokine levels were observed in concordance with known *in vivo* infection dynamics [7].

Beyond pathogen colonization and invasion, GoC systems have proven useful for studying toxin-mediated pathogenic mechanisms. A recent study by Meza-Torres et al. [69] evaluated a *Clostridium difficile* epidemic strain (CDT) and generated a mutant lacking toxins A and B (CDT-). Results obtained in the GoC showed that the CDT induced mucin-associated microcolonies, enhancing its colonization and promoting biofilm-like properties. This GoC model recapitulates observations *in vivo*, as these biofilm-like microcolonies were also observed in the cecum and colon of infected mice.

GoC technology has also emerged as a valuable tool for studying bacteriophages and their interactions with the intestinal mucosa. In the context of phage therapy, studies by Wu et al. [64] and Van Nieuwenhuyse et al. [61] demonstrated that GoC platforms–containing either HT-29 or mixed Caco-2/LS174T cells—possess unique advantages for modeling phage-mediated decontamination. These platforms offer the ability to observe real-time phage–bacteria dynamics under physiologically relevant conditions.

Although viral immune responses are most comprehensively studied in whole-animal systems, GoC models have proven highly effective for recapitulating human-specific enteric viral infections that do not readily infect non-human hosts. Villenave et al. [34] modelled coxsackievirus B1 infection by administering virus through either the apical or basal channel. The platform allowed detailed characterization of viral entry, replication, and cytokine induction. Basal-route infection led to reduced viral titers and delayed caspase-3 activation compared to apical infection, while cytokine secretion remained unaltered, highlighting route-specific differences in host responses.

In addition, GoCs have also been employed to investigate enteric manifestations of coronavirus infection. Guo et al. [19]’s study demonstrated that GoC expressed high levels of ACE2, which facilitates its infection by SARS-CoV-2 coronavirus. Infected GoC exhibited significant epithelial barrier damage, heightened cytokine release, immune cell recruitment and endothelial inflammation, which recapitulates some hallmark features of coronavirus-induced intestinal pathology.

Beyond bacteria and viruses, GoC devices are now being applied to study fungal pathogens such as *Candida albicans*, a commensal yeast capable of causing systemic infections under dysbiotic or immunocompromised conditions. Kaden et al. [58] modelled early candidiasis in GoC, showing that *C. albicans* microcolonies disrupt the epithelial barrier and trigger inflammatory responses. Moreover, treatment with caspofungin significantly reduced fungal biomass and altered hyphal morphology, demonstrating the utility of GoC for antifungal drug evaluation.

In a complementary study, Maurer et al. [44] also investigated *C. albicans* infection in the presence of *L. rhamnosus* ATCC 7469. The results indicated that the probiotic strain inhibited *C. albicans* colonization and prevented its translocation across the epithelial barrier, highlighting GoC applications for studying microbe–microbe interactions and its protective mechanisms.

In summary, GoC platforms have become powerful tools for modelling diverse intestinal infections by enabling detailed examination of host–microbe interactions under physiologically relevant conditions. Collectively, the studies discussed above demonstrate the capacity of these systems to reproduce selected aspects of human pathogenesis, including epithelial barrier disruption, cytokine and chemokine responses, microbial virulence behaviours, and tissue-specific infection dynamics. Their versatility is demonstrated by their ability to distinguish commensal from pathogenic organisms, capture strain-specific and route-specific infection dynamics, and model phage, fungal, and toxin-induced processes. Importantly, GoC platforms facilitate controlled host–microbe and microbe–microbe interactions, making them valuable tools for evaluating therapeutic and biotic interventions. Taken together with the advancements described in earlier sections, such as patient-derived tissues, microbiota-integrated systems, and disease-specific organoid-chip platforms, these findings underscore the broad and transformative potential of GoC technology across multiple dimensions of gastrointestinal research.

## 4. Discussion

This systematic review provides a comprehensive overview of host–microbiome interaction studies using GoC models, including investigations involving probiotics, postbiotics, commensal bacteria, pathogenic microorganisms, and faecal-derived microbiota.

One of the first challenges encountered during the systematic review process was the taxonomic reclassification of the genus *Lactobacillus* into 25 distinct genera in 2020 [72]. Our search strategy included only the term *Lactobacillus*, and incorporating all newly established genera would have substantially increased the complexity of the search process. To assess the potential impact of this taxonomic revision, we evaluated whether inclusion of the newly established genera would identify additional eligible studies. This assessment did not retrieve any additional records beyond those identified through the original search strategy. This is further supported by the inclusion of complementary search terms, such as probiotic and microbiota, and by the use of three independent bibliographic databases, which together helped maximize the retrieval of relevant studies. Therefore, based on the results of this assessment, the taxonomic revision was not found to influence study identification in the present review.

Regarding the GoC technologies, despite their rapid advancement and growing adoption across universities and research institutes—including dedicated facilities such as the Centre for Predictive *In Vitro* Models at Queen Mary University of London—important limitations still constrain their broader implementation in host–biotic–microbiome research.

First, generating clinically meaningful microbiome-based studies requires ethics-approved patient recruitment, yet strict inclusion criteria and the need for unequivocal diagnostic documentation frequently make the enrolment of sufficiently powered cohorts challenging.

A second major limitation lies in the technical difficulty of sustaining stable co-cultures of human intestinal cells with complex microbiota under physiologically relevant oxygen gradients. Conventional incubators cannot simultaneously maintain the hypoxic luminal environment required for anaerobes and the oxygen-rich basolateral conditions needed for epithelial viability.

Furthermore, the microbiome models identified in this review encompass different levels of microbial complexity, ranging from single-strain cultures and defined microbial consortia to whole faecal microbiota communities. While single-strain systems facilitate mechanistic investigations under highly controlled conditions, defined consortia allow the study of selected microbial interactions. In contrast, whole faecal microbiota models more closely resemble the complexity of the intestinal ecosystem but introduce additional challenges related to stability, community composition, and reproducibility.

Although several platforms have successfully cultured strict anaerobic probiotic strains using deoxygenated media, recreating stable, diverse microbial communities remains challenging. Oxygen diffusion into the apical compartment often leads to overgrowth of facultative aerobes and underrepresentation of strict anaerobes, thereby reducing ecological fidelity and limiting interpretation of microbiome-driven effects.

Reproducibility is another significant concern in this emerging field. Technical variability may arise from differences in chip materials, device geometries, membrane characteristics, flow regimes, oxygen-control strategies, and analytical workflows across platforms. In addition, biological variability, including differences between cell donors and variations in microbial community composition, can substantially affect experimental outcomes and complicate cross-study comparisons. The high cost of equipment, specialized consumables, and extended experimental durations—often spanning several weeks—can restrict the number of biological and technical replicates achievable in typical laboratory settings, thereby reducing statistical power.

Standardization across platforms is also limited, as device materials, geometries, flow regimes, and analytical workflows vary widely between manufacturers. Recognizing these gaps, international standard-setting bodies—including the European Commission and ISO—have launched initiatives such as the ISO/TC 276/SC2 Subcommittee on Microphysiological Systems to develop harmonized guidelines. To facilitate reproducibility and cross-platform comparisons, future GoC studies would benefit from reporting a minimum set of experimental parameters, including chip material and dimensions, membrane composition and characteristics, cell source and donor information, flow rate and estimated shear stress, oxygen tension and oxygen-control strategy, microbial inoculum composition and concentration, culture duration and sampling time points, analytical methods, and the numbers of biological and technical replicates. In addition, comparative cross-platform studies assessing epithelial gene expression, barrier function, tight junction protein profiles, mucus production, cytokine responses, and biotic functionality will be essential for validating equivalence and ensuring reproducibility.

Beyond these technical and standardization challenges, an additional knowledge gap concerns the limited use of GoC systems for postbiotic assessment. To date, most studies involving biotics in GoC models have been conducted using probiotic strains, defined microbial consortia, or faecal microbiota-derived communities. By contrast, postbiotics remain comparatively underexplored as their functionality has rarely been evaluated in these systems. GoC platforms may be particularly well suited for this purpose because they provide highly controlled experimental environments that enable the evaluation of host responses to microbial-derived products independently of microbial colonization, growth, and interspecies interactions. This may facilitate the distinction between effects directly associated with live microorganisms and those mediated by microbial metabolites or other bioactive compounds. This highlights an important future research direction, as this technology could provide a valuable platform to investigate the mechanisms of action, safety, and host responses associated with postbiotic interventions.

Therefore, it is expected that the coming years will see an increase in studies exploring the effects of postbiotics in GoC models.

Despite the substantial progress achieved in recent years, several challenges continue to limit the broader implementation of GoC models in host–microbiome research. In particular, improvements in oxygen control, maintenance of complex microbial communities, reproducibility, and platform standardization remain necessary. Addressing these challenges through technological refinement, harmonized reporting practices, and cross-platform validation studies will be essential to enhance the robustness and comparability of future investigations.

## 5. Conclusions

**Figure 2:**
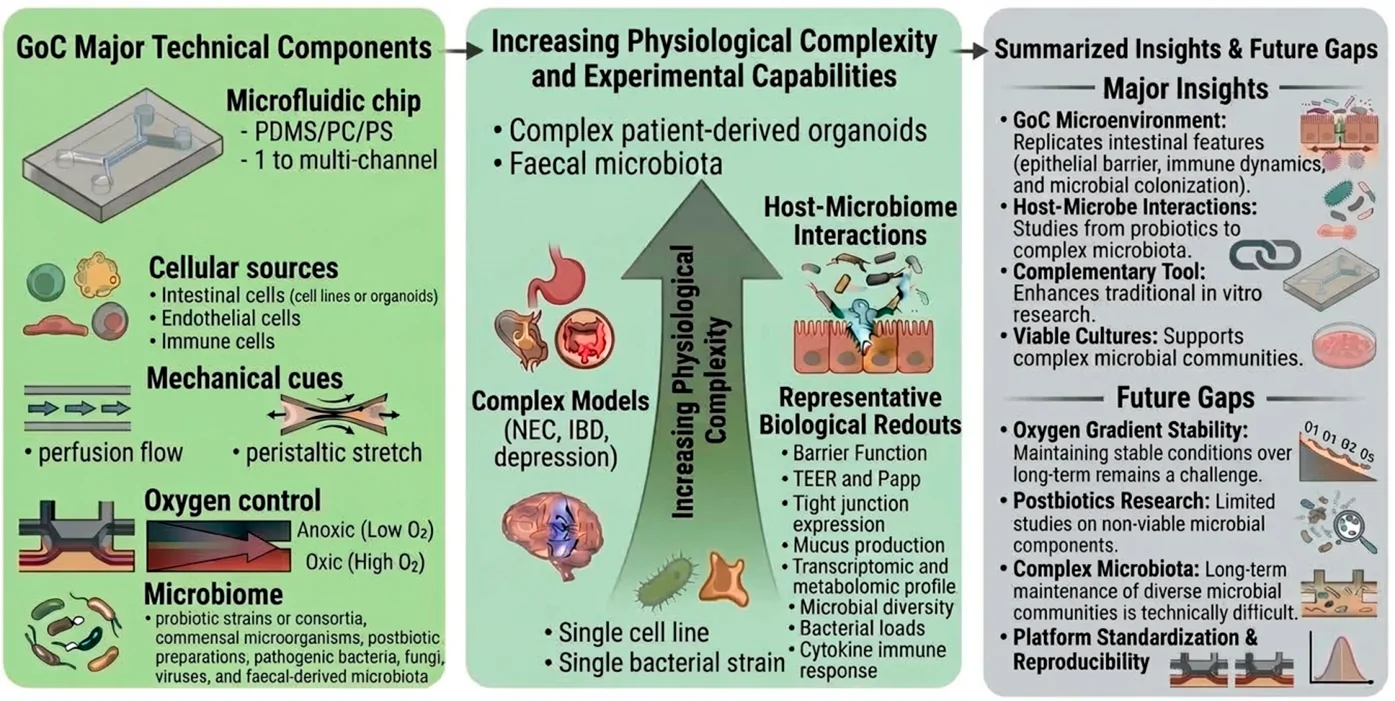
Overview of the conceptual framework, key findings, and future opportunities in GoC host microbiome research. PDMS: Polydimethylsiloxane; PC: polycarbonate; PS: polystyrene; NEC: necrotizing enterocolitis; IBD: irritable bowel disease; TEER: transepithelial electrical resistance; Papp: apparent permeability.

This systematic review highlights the growing use of gut-on-chip (GoC) technologies for investigating host–microbiome interactions under physiologically relevant conditions. The available evidence demonstrates that these platforms can reproduce selected aspects of intestinal physiology and host–microbe interactions, including epithelial barrier function, immune responses, microbial colonization, and the effects of probiotic, commensal, pathogenic, and microbiota-derived communities. As such, GoC models represent valuable tools that complement conventional *in vitro* approaches and enable mechanistic investigations of the intestinal ecosystem (Figure 2).

However, significant challenges remain. The ability to maintain physiologically relevant oxygen gradients, support complex microbial communities, ensure reproducibility, and achieve cross-platform standardization continues to limit physiological fidelity and direct comparisons between studies. Addressing these challenges through technological refinement, harmonized reporting practices, and cross-platform validation will be essential to improve the robustness and translational relevance of future investigations.

Current evidence primarily supports the application of GoC systems to studies involving live microorganisms and microbiota-derived communities, whereas postbiotic research remains limited. Consequently, the use of GoC platforms for postbiotic assessment should be regarded as an emerging research opportunity rather than an established application. Continued technological development and standardization efforts will be necessary to determine the extent to which these systems can support future biomedical, nutritional, and therapeutic applications.

## Data Availability

No new data were created or analysed in this study. Data sharing is not applicable to this article.

## Supplementary Materials

The following supporting information can be downloaded at https://www.mdpi.com/article/10.3390/microorganisms14092059/s1: Table S1: Modified SYRCLE’s Risk of Bias tool; Table S2: Risk of bias (RoB) analysis for *in vitro* studies (Modified SYRCLE’s RoB tool). References [26,73] are cited in the Supplementary Material.

## Abbreviations

The following abbreviations are used in this manuscript:

5-HT	Serotonin
ATCC	American Type Culture Collection
CDT	*Clostridium difficile* epidemic strain
DAMP	Damage-associated molecular patterns
DARPA	Defense Advanced Research Projects Agency
DMSZ	German Collection of Microorganisms and Cell Cultures
EHEC	Enterohemorrhagic *Escherichia coli*
EIEC	Enteroinvasive *Escherichia coli*
FDA	Food and Drug Administration
GABA	Gamma-aminobutyric acid
GoC	Gut-on-chip
HIMEC	Human intestinal microvascular endothelial cell
HUVEC	Human umbilical vein endothelial cell
IBS	Irritable bowel syndrome
IL	Interleukin
LPS	Lipopolysaccharide
NEC	Necrotizing enterocolitis
NIH	National Institutes of Health
OoC	Organ-on-chip
PBMC	Peripheral blood mononuclear cells
PC	Polycarbonate
PDMS	Polydimethylsiloxane
PRISMA	Preferred reporting items for systematic reviews and meta-analyses
PS	Polystyrene
RoB	Risk of bias
SARS-CoV-2	Severe acute respiratory syndrome-related coronavirus-2
SCFA	Short chain fatty acid
SIBO	Small intestinal bacterial overgrowth
TEER	Transepithelial electrical resistance
TNF-α	Tumor necrosis factor-alpha
