# Supplementary material for: Gut-on-Chip Models for Host–Microbiome Studies: A Systematic Review
Source: Microorganisms. 2026 Sep 15;14(9):2059. doi: 10.3390/microorganisms14092059 (PMC13609488; doi:10.3390/microorganisms14092059)
Supplement: Supplementary file 1 [file microorganisms-14-02059-s001.zip › microorganisms-4531702-supplementary.pdf]

Supplementary Materials

Table S1. Modified SYRCLE's Risk of Bias (RoB) tool

Table S2. RoB analysis for *in vitro* studies (Modified SYRCLE's RoB tool)

Modified SYRCLE's RoB tool. The SYRCLE's RoB tool, designed to assess bias in in vitro research (De la Rosa González et al. 2024) was further adapted. Selection bias was reformulated to highlight possible differences between the chosen cell lines whether they were immortalized, primary cell cultures, biopsies or organoids. And additional selection bias was included to describe the nature of microbiome intervention, specifying whether it consists of an entire fecal-derived microbial community or defined elements (e.g., strains, microbial consortia, or selected microbial components) The performance bias were reformulated to highlight the variability of the interventions. For detection bias, reporting bias and others we used the domains and descriptions used by De la Rosa González et al. (2024). Finally, attrition bias was deleted from the RoB tool. The resulting RoB tool for *in vitro* studies included in our review contains 10 entries, as shown in Table S1.

Table S1. Modified SYRCLE's Risk of Bias (RoB) tool

Item	Type of bias	Domain	Description of domain	Review authors judgment
1	Selection bias	Sample size calculation	Details of the sample size calculation method (software, formula and parameters used to calculate the sample size) should be clearly indicated	Was the sample size properly calculated and applied? (a)
2	Selection bias	Baseline characteristics	Details of cell lines used	Is it clear whether the cell lines used are immortalized, primary cell cultures, biopsies or organoids? (b)
3	Selection bias	Baseline characteristics	Details of microbiome (e.g. probiotic, bacteria, phage, virus, fecal samples) used	Does the study clearly describe the nature of microbiome intervention, specifying whether it consists of an entire fecal-derived microbial community or defined elements (e.g., strains, microbial consortia, or selected microbial components)? (c)
4	Performance bias	Detailed explanation of intervention	The intervention procedure must be clearly stated, describing the concentration, and the incubation time of the experiment	Were interventions administered to cells in a consistent and standardized manner throughout the experiment? (b)
5	Performance bias	Detail explanation of culture conditions	Provide any information relating to culture conditions of chip (flow, vacuum)	Are culture conditions comparable between different studies or groups within studies? (b)
6	Detection bias	Details of comparison group	Details of comparison group (positive control, negative control, or standard) should be clearly specified.	Were appropriate controls included? (a)
7	Detection bias	Outcome measuring method	The procedure and rationale for the choice of method, and how the results were evaluated should be clearly stated	Were the statistical analysis clearly specified? (a)
8	Detection bias	Blinding	Details on all measures used, if any, to prevent outcome assessors from knowing which intervention each cell culture received. Provide any information regarding the effectiveness of the planned blinding.	Was the outcome assessor blinded? (d)
9	Reporting bias	Selective outcome reporting	State how selective outcome reporting was examined and what was found.	Are reports of the study free of selective outcome reporting? (d)
10	Other	Other sources of bias	State any important concerns about bias not covered by other domains in the tool.	Was the study apparently free of other problems that could result in high risk of bias? (d)

a Item in agreement with the SYRCLE´s tool for assessing risk of bias from in vitro studies (De la Rosa González et al. Foods. 2024, 13(21):3479)
b Item reformulated from SYRCLE´s tool for assessing risk of bias from in vitro studies (De la Rosa González et al. Foods. 2024, 13(21):3479)
c Item included in the SYRCLE´s tool for assessing risk of bias from in vitro studies.
d Items in agreement with the SYRCLE's tool for assessing risk of bias from animal studies (Hooijmans et al. BMC Medical Research Methodology 2014, 14:43)

Table S2. RoB analysis for *in vitro* studies (Modified SYRCLE's RoB tool).

Study	Item 1	Item 2	Item 3	Item 4	Item 5	Item 6	Item 7	Item 8	Item 9	Item 10
Kim et al. (2012)	No	Yes	Yes	Yes	Yes	Yes	Yes	No	Yes	Yes
Marzorati et al. (2014)	No	Yes	Yes	Yes	Yes	Yes	Yes	No	Yes	Yes
Shah et al. (2016)	No	Yes	Yes	Yes	Yes	Yes	Yes	No	Yes	Yes
Kim et al. (2016)	No	Yes	Yes	Yes	Yes	Yes	Yes	No	Yes	Yes
Villenave et al. (2017)	No	Yes	Yes	Yes	Yes	Yes	Yes	No	Yes	Yes
Shin and Kim. (2018)	No	Yes	Yes	Yes	Yes	Yes	Yes	No	Yes	Yes
Grassart et al. (2019)	No	Yes	Yes	Yes	Yes	Yes	Yes	No	Yes	Unclear
Greenhalgh et al. (2019)	No	Yes	Yes	Yes	Yes	Yes	Yes	No	Yes	Yes
Jalili-Firoozinezhad et al. (2019)	Yes	Yes	Yes	Yes	Yes	Yes	Yes	No	Yes	Yes
Maurer et al. (2019)	No	Yes	Yes	Yes	Yes	Yes	Yes	No	Yes	Yes
Shin et al. (2019)	No	Yes	Yes	Yes	Yes	Yes	Yes	No	Yes	Yes
Tovaglieri et al. (2019)	No	Yes	Yes	Yes	Yes	Yes	Yes	No	Yes	Yes
Jing et al. (2020)	No	Unclear	Yes	Yes	Yes	Yes	Yes	No	Yes	Yes
Shin et al. (2020)	No	Yes	Yes	Yes	Yes	Yes	Yes	No	Yes	Yes
Yuan et al. (2020)	No	Yes	Yes	Yes	Yes	Yes	Yes	No	Yes	Yes
Gazzaniga et al. (2021)	No	Yes	Yes	Yes	Yes	Yes	Yes	No	Yes	Yes
Guo et al. (2021)	No	Unclear	Yes	Unclear	Unclear	Yes	Yes	No	Yes	Yes
Nelson et al. (2021)	No	Yes	Yes	Yes	Yes	Yes	Yes	No	Yes	Yes
Chin et al. (2022)	No	Yes	No	Yes	Yes	Yes	Yes	No	Yes	Yes
De Gregorio et al. (2022)	No	Unclear	Yes	Yes	Yes	Yes	Yes	No	Yes	Yes
Jeon et al. (2022)	No	Yes	Yes	Yes	Yes	Yes	Yes	No	Yes	Yes
Jing et al. (2022)	No	Yes	Yes	Yes	Yes	Yes	Yes	No	Yes	Yes
Lee et al. (2022)	No	Unclear	No	No	Yes	Yes	No	No	Yes	Yes
Min et al. (2022)	No	Yes	Yes	Yes	Yes	Yes	Unclear	No	Yes	Yes
Zhao et al. (2022)	No	Unclear	Unclear	Yes	Yes	Yes	Yes	No	Yes	Yes
Lanik et al. (2023)	No	Yes	Yes	Unclear	Yes	Yes	Yes	No	Yes	Yes
Liu et al. (2023)	No	No	Yes	Yes	Yes	Yes	Unclear	No	Yes	Yes
Nelson et al. (2023)	No	Yes	Yes	Yes	Yes	Yes	Yes	No	Yes	Yes
Donkers et al. (2024)	No	Yes	Yes	No	No	Yes	Yes	No	Yes	Yes
Kaden et al. (2024)	No	Yes	Yes	Yes	Yes	Yes	Yes	No	Yes	Yes
Lee et al. (2024)	No	Yes	Yes	Yes	Yes	Yes	Yes	No	Yes	Yes
Lucchetti et al. (2024)	No	Yes	Yes	Unclear	Yes	Yes	Yes	No	Yes	Yes
Van Nieuwenhuyse et al. (2024)	No	Yes	Yes	Yes	Yes	Yes	Yes	No	Yes	Yes
Wang et al. (2024)	No	Yes	Yes	Unclear	Yes	Yes	Yes	No	Yes	Yes
Wu et al. (2024a)	No	Yes	Yes	Yes	Unclear	Yes	Yes	No	Yes	Yes

Wu et al. (2024b)	No	Yes	Unclear	Yes	Yes	Yes	Yes	No	Yes	No
Ballerini et al. (2025)	No	Unclear	Yes	No	No	Yes	Yes	No	Yes	Yes
Belotserkovsky et al. (2025)	No	Yes	Yes	Yes	Yes	Yes	Yes	No	Yes	Yes
Canourgues et al. (2025)	No	Yes	Yes	Yes	Yes	Yes	Yes	No	Yes	Yes
Dash et al. (2025)	No	Unclear	Yes	Yes	Unclear	Yes	Yes	No	Yes	Yes
Delannoy et al. (2025)	No	Yes	Yes	Yes	Yes	Yes	No	No	Yes	Yes
Lange et al. (2025)	No	Yes	Yes	Yes	No	Yes	Yes	No	Yes	Yes
Lee et al. (2025)	No	Unclear	Yes	Yes	Yes	Yes	Yes	No	Yes	Yes
Meza-Torres et al. (2025)	No	Yes	Yes	Yes	Yes	Yes	Yes	No	Yes	Yes
Clayton et al. (2026)	No	Unclear	Yes	Yes	Yes	Yes	Yes	No	Yes	Yes
De Rudder et al. (2026)	No	Yes	Yes	Yes	Yes	Yes	Yes	No	Yes	Yes
Li et al. (2026)	No	Yes	Yes	Yes	Yes	Yes	Yes	No	Yes	Yes
Xiang et al. (2026)	No	Yes	Unclear	Unclear	No	Yes	Yes	No	Yes	Yes

References

Hooijmans CR, Rovers MM, de Vries RB, Leenaars M, Ritskes-Hoitinga M, Langendam MW. SYRCLE's risk of bias tool for animal studies. BMC Med Res Methodol. 2014 Mar 26;14:43. doi: 10.1186/1471-2288-14-43. PMID: 24667063; PMCID: PMC4230647.

De la Rosa González A, Guerra-Ojeda S, Camacho-Villa MA, Valls A, Alegre E, Quintero-Bernal R, Martorell P, Chenoll E, Serna-García M, Mauricio MD, Serna E. Effect of Probiotics on Gastrointestinal Health Through the Aryl Hydrocarbon Receptor Pathway: A Systematic Review. Foods. 2024 Oct 30;13(21):3479. doi: 10.3390/foods13213479. PMID: 39517263; PMCID: PMC11545787.
